# Supplementary material for: Chromothripsis during telomere crisis is independent of NHEJ, and consistent with a replicative origin
Source: Genome Res. 2019 May;29(5):737–49. doi: 10.1101/gr.240705.118 (PMC6499312; doi:10.1101/gr.240705.118)
Supplement: Supplemental Material [file supp_gr.240705.118_Supplemental_file_1.zip › contigs/annotated_contigs/DB51/contig.2.DB51_length_578_mean_cov_4.3875432526.docx]

**DB51_length_578_mean_cov_4.3875432526**

```
TTTGGGGCACAATTCCATGACCACCTACTGACTGTGAGGTTTGTCAGCCACACAGAAGAATAAAAAAAGGCACGTAAGACTTGAATTAG
 >chr20:22198119-22198442 - E=1e-176
CATGGTGTGAAAACAAAAGTCAGTAAAAAGAGCCCGGGTGTGAGGAGGTATGAAAATGAAAACATGTTCTGCAAGAAAAGGGGTTTTTG

ATATGCTGAGAGACAAGTGCTGGGGAAATTTAAATTCCCATCTTTTACCAAGCAGCAACCCTGATTTTTATTGCACTTGCTGTTTAAAG

AATTCAACAAGCCTCAGTCCTTTAACTCTAACAGTCTCCAGCTGACATCATGTGAA|TCAA|AACAGTTCATAAAGTCAGCATTCATCC
                                                             >chr20:10864659-10864910 -
CGCTGATAATTTTAGGCAGTGCCCAGTTAACATTCAATCTGTGTTATAAACAATTGCTACTGTGTGTTGTTAATACCACTGGGGACAAA
 E=2e-136
AGGTGCAGCTCTTACAACAGTGATCCAGGAGTCCTTGGATTAATTGACAAGGACTCATGGATTGATTTTCACACCAAGCTTTATCTATA

AACATATATTAATAATATGTTTCATACAAATATATACCCATAACAA
```
